# Dm5-HT_2B_: Pharmacological Characterization of the Fifth Serotonin Receptor Subtype of *Drosophila melanogaster*

**DOI:** 10.3389/fnsys.2017.00028

**Published:** 2017-05-11

**Authors:** Wolfgang Blenau, Stöppler Daniel, Sabine Balfanz, Markus Thamm, Arnd Baumann

**Affiliations:** ^1^Cologne Biocenter and Zoological Institute, University of CologneCologne, Germany; ^2^Department of NMR-Supported Structural Biology, Leibniz-Institut für Molekulare PharmakologieBerlin, Germany; ^3^Institute of Complex Systems – Cellular Biophysics (ICS-4), Forschungszentrum JülichJülich, Germany; ^4^Behavioral Physiology and Sociobiology (Zoology II), Biocenter, University of WürzburgWürzburg, Germany

**Keywords:** biogenic amine, Ca^2+^, cAMP, cellular signaling, insect, G protein-coupled receptor, inositol-1, 4, 5-trisphosphate, second messenger

## Abstract

Serotonin (5-hydroxytryptamine, 5-HT) is an important regulator of physiological and behavioral processes in both protostomes (e.g., insects) and deuterostomes (e.g., mammals). In insects, serotonin has been found to modulate the heart rate and to control secretory processes, development, circadian rhythms, aggressive behavior, as well as to contribute to learning and memory. Serotonin exerts its activity by binding to and activating specific membrane receptors. The clear majority of these receptors belong to the superfamily of G-protein-coupled receptors. In *Drosophila melanogaster*, a total of five genes have been identified coding for 5-HT receptors. From this family of proteins, four have been pharmacologically examined in greater detail, so far. While Dm5-HT_1A_, Dm5-HT_1B_, and Dm5-HT_7_ couple to cAMP signaling cascades, the Dm5-HT_2A_ receptor leads to Ca^2+^ signaling in an inositol-1,4,5-trisphosphate-dependent manner. Based on sequence similarity to homologous genes in other insects, a fifth *D. melanogaster* gene was uncovered coding for a Dm5-HT_2B_ receptor. Knowledge about this receptor’s pharmacological properties is very limited. This is quite surprising because Dm5-HT_2B_ has been attributed to distinct physiological functions based on genetic interference with its gene expression. Mutations were described reducing the response of the larval heart to 5-HT, and specific knockdown of Dm5-HT_2B_ mRNA in hemocytes resulted in a higher susceptibility of the flies to bacterial infection. To gain deeper understanding of Dm5-HT_2B_’s pharmacology, we evaluated the receptor’s response to a series of established 5-HT receptor agonists and antagonists in a functional cell-based assay. Metoclopramide and mianserin were identified as two potent antagonists that may allow pharmacological interference with Dm5-HT_2B_ signaling *in vitro* and *in vivo*.

## Introduction

The biogenic amine serotonin (5-hydroxytryptamine, 5-HT) is an ancient neuroactive substance and present throughout the animal kingdom. Serotonin plays a key role in regulating and modulating many physiological and behavioral processes in both protostomes and deuterostomes. In humans, malfunction of the serotonergic system has been associated with several impairments and diseases, such as schizophrenia, migraine, depression, suicidal behavior, infantile autism, eating disorders, and obsessive-compulsive disorder (for reviews, Green, 2006; Geyer and Vollenweider, 2008; Berger et al., 2009).

To gain insight into serotonergic function(s), insects are highly attractive models. In comparison with vertebrates and especially mammals, they allow assessing the anatomical distribution, development, and neurophysiological properties of serotonergic neurons with unprecedented inter-individual reproducibility and precision. Applying this experimental strategy, the activity of serotonergic neurons has been related to physiological functions and changes in behavior (for reviews, Walz et al., 2006; Blenau and Thamm, 2011; Ellen and Mercer, 2012; Nall and Sehgal, 2014; Vleugels et al., 2015). In fruit flies (*Drosophila melanogaster*), certain behavioral effects have been ascribed to the serotonergic system. In *D. melanogaster* larvae, serotonin modulates the heart rate (Dasari and Cooper, 2006) and is involved in olfactory processing (Python and Stocker, 2002), feeding behavior (Neckameyer et al., 2007; Neckameyer, 2010), locomotion (Majeed et al., 2016), and responses to light (Rodriguez Moncalvo and Campos, 2009). In adult flies, serotonergic neurons participate in the regulation of nutrient balance (Vargas et al., 2010; Ro et al., 2016), insulin signaling and organismal growth (Kaplan et al., 2008; Luo et al., 2012, 2014), locomotion (Neckameyer et al., 2007; Majeed et al., 2016), olfactory processing (Dacks et al., 2009), aggression (Dierick and Greenspan, 2007; Alekseyenko et al., 2010, 2014; Alekseyenko and Kravitz, 2014), circadian rhythm (Yuan et al., 2005), sleep (Yuan et al., 2006), courtship and mating behavior (Becnel et al., 2011), and learning (Sitaraman et al., 2008, 2012; Lee et al., 2011).

The diverse cellular and behavioral effects of serotonin in insects are mediated by a family of G protein-coupled receptors (GPCRs). So far, four 5-HT receptor subtypes have been pharmacologically characterized in *D. melanogaster*. These are Dm5-HT_1A_ and Dm5-HT_1B_ (CG16720 and CG15113; Saudou et al., 1992), Dm5-HT_2A_ (CG1056; Colas et al., 1995), and Dm5-HT_7_ (CG12073; Witz et al., 1990). These GPCRs are assumed to be orthologs of mammalian 5-HT_1A_, 5-HT_2_, and 5-HT_7_ receptors. Due to sequence homology with a 5-HT_2_ receptor in the spiny lobster, *Panulirus interruptus*, a second 5-HT_2_ receptor gene (CG42796) has been postulated in *D. melanogaster* (Clark et al., 2004). This observation was corroborated by independent bioinformatics studies (Hauser et al., 2006; Blenau and Thamm, 2011) and was substantiated experimentally by molecular cloning (Gasque et al., 2013). The receptor was named Dm5-HT_2B_. Although orthologous receptors have been characterized in other insects as well, e.g., the honeybee *Apis mellifera* (Thamm et al., 2013) and the kissing bug *Rhodnius prolixus* (Paluzzi et al., 2015), knowledge about the pharmacological properties of Dm5-HT_2B_ is rather limited. This is quite surprising because Dm5-HT_2B_ has been attributed to distinct physiological functions. For example, Dm5-HT_2B_ receptor mutations reduce the response of the larval heart to 5-HT (Majeed et al., 2014). Furthermore, knockdown of Dm5-HT_2B_ gene expression by RNAi in hemocytes caused reduced phagocytotic clearance and thus resulted in a higher susceptibility of the flies to bacterial infection (Qi et al., 2016). At the behavioral level, it has been uncovered that reducing the level of Dm5-HT_2B_ expression by either RNAi or transposon insertion into the gene locus leads to a decrease in anxiety-like behavior (Mohammad et al., 2016).

The aim of the current study was to focus on the pharmacological properties of the Dm5-HT_2B_ receptor. The cDNA encoding Dm5-HT_2B_ was amplified on mRNA extracted from *D. melanogaster* heads. A cell line was established constitutively expressing Dm5-HT_2B_. Since 5-HT_2B_ receptors are known to cause inositol-1,4,5-trisphosphate (IP_3_)-mediated Ca^2+^ release from intracellular stores, we examined Dm5-HT_2B_ functionality by Ca^2+^ fluorimetry. The receptor’s pharmacological profile was established after applying concentration series of various agonists and antagonists. In addition to serotonin as the native ligand, 5-methoxytryptamine and 8-Hydroxy-2-(di-n-propylamino)tetralin (8-OH-DPAT) were very potent agonists. Receptor activity was efficiently blocked by metoclopramide and mianserin. Thus, this study provides important new data regarding the pharmacological characteristics of the fifth 5-HT receptor of the fruit fly.

## Materials and Methods

### Cloning of the *Dm5-ht2b* cDNA

Poly(A)^+^ RNA was prepared from 180 heads of male flies (*D. melanogaster*, *w^*1118*^*) by using the Micro-Fast Track^TM^ 2.0 Kit (Invitrogen, Karlsruhe, Germany). Synthesis of cDNA employed the AccuScript^TM^ High Fidelity First Strand cDNA Synthesis Kit (Stratagene, Amsterdam, Netherlands). For amplification of the entire coding region of *Dm5-ht2b*, specific primers were designed based on available sequence information (Brody and Cravchik, 2000; Clark et al., 2004; Hauser et al., 2006; Gasque et al., 2013): sense primer 5′-CAGAGTAGAGCGCACAATAGG-3′ (position -35 to -15); antisense primer 5′-GTTTGCCCGGTTTAACG-3′ (position 2724 to 2740; TIB Molbiol, Berlin, Germany). The polymerase chain reaction (PCR) was carried out for 30 s at 98°C (1 cycle) followed by 35 cycles of 10 s at 98°C, 30 s at 62°C, 90 s at 72°C, and a final extension of 10 min at 72°C. The reaction was performed with Phusion^®^ High Fidelity DNA Polymerase (New England Biolabs, Frankfurt am Main, Germany). PCR products were cloned into pGEM-T vector (Promega, Mannheim, Germany) and subsequently sequenced (GATC Biotech AG, Konstanz, Germany).

### Multiple Sequence Alignments and Phylogenetic Analysis

Amino-acid sequences used for phylogenetic analysis were identified by protein-protein Basic Local Alignment Search Tool (BLAST) searches of the National Center for Biotechnology Information (NCBI) database with the deduced amino acid sequence of *Dm5-ht2b* (Dm5-HT_2B_) as “bait.” Values for identity (ID) and similarity (S) were calculated by using the BLOSUM62 substitution matrix in BioEdit 7.1.9. Phylogenetic analysis was conducted as described by Reim et al. (2017) using Bayesian analysis (MrBayes v.3.2.6; Ronquist et al., 2012) with the substitution model LG +G, determined by Protest 3.4.2 (Darriba et al., 2011). Human rhodopsin (HsRHOD) and *D. melanogaster* FMRFamide receptor (DmFMRFaR) sequences were used to root the phylogenetic tree.

### Construction of Expression Vectors

An expression-ready construct of *Dm5-ht2b* was generated in pcDNA3.1 vector (Invitrogen/ThermoFisher Scientific, Darmstadt, Germany). PCR was performed with specific primers (sense primer: 5′-AATAAGCTT*CCACC*ATGGAAGAGGATGTGTATGCC-3′; antisense primer first-round PCR: 5′-TGGGACGTCGTATGGGTATCTGCTCGGTCGCCAGG-3′; antisense primer second-round PCR: 5′-TTTTCTAGACTCGAGTTAAGCGTAGTCTGGGACGTCGTATGGGTA-3′). PCR products were digested with HindIII and XhoI, and subcloned into pcDNA3.1(+) vector (Invitrogen). Thus, the resulting construct contained the Kozak consensus motif (CCACC, Kozak, 1984) immediately 5′ to the ATG-codon and a hemagglutinin A (HA) epitope tag (amino acid sequence: YPYDVPDYA) at the 3′ end of the *Dm5-ht2b* cDNA and was named pc*Dm5-ht2b*-HA. The insert fragment was checked by DNA sequencing.

### Functional Expression in Mammalian Cell Lines

Approximately 8 μg of pc*Dm5-ht2b*-HA was transfected into exponentially growing HEK 293 cells (∼4 × 10^5^ cells per 5-cm Petri dish) by a modified calcium phosphate method (Chen and Okayama, 1987). Stably transfected cells were selected in the presence of the antibiotic G418 (0.8 mg/ml). Isolated foci were propagated and analyzed for the expression of Dm5-HT_2B_-HA receptor either by immunocytochemistry, Western blotting or by functional Ca^2+^ imaging upon receptor activation.

### Functional and Pharmacological Characterization of Dm5-HT_2B_

The ability of the Dm5-HT_2B_-HA receptor (hereafter referred to as Dm5-HT_2B_) to activate G_q_ proteins was assessed by monitoring changes in [Ca^2+^]_i_ with the Ca^2+^-sensitive fluorescent dye Fluo-4 (Invitrogen). Non-transfected HEK 293 cells and cells expressing Dm5-HT_2B_ were grown in minimal essential medium (MEM + GlutaMAX^TM^I (Gibco/ThermoFisher Scientific, Darmstadt, Germany) containing 2% (w/v) Utroser^TM^ G (Pall, Dreieich, Germany), 1 × non-essential amino acids and 1 × antibiotics/antimycotics) in 96-well plates to a density of ∼3 × 10^4^ cells per well. In this format, each vertical row (=8 wells) of the 96-well plate is incubated with the same ligand concentration. Cells were loaded at room temperature with Fluo-4 as described earlier (Thamm et al., 2013; Blankenburg et al., 2015) in extracellular solution [ES = in mM: 120 NaCl, 5 KCl, 2 MgCl_2_, 2 CaCl_2_, 10 HEPES, 10 Glucose, pH 7.4 (NaOH)]. Plates were transferred into a fluorescence reader (FLUOstar Galaxy/Optima; BMG Labtech, Offenburg, Germany) to monitor Fluo-4 fluorescence. The excitation wavelength was 485 nm, and fluorescence emission was detected at 520 nm. Various concentrations of biogenic amines and synthetic receptor ligands were added, once Fluo-4 fluorescence had reached a stable value in each well. The changes in Fluo-4 fluorescence were recorded automatically. Concentration-response curves for putative agonists/antagonists were established in at least two independent experiments with octuplicate determinations (s.a.) per data point. Data were analyzed and displayed by using PRISM 5.0.4 software (GraphPad, San Diego, CA, USA).

## Results

### Cloning of *Dm5-ht2b* cDNA and Structural Properties of Dm5-HT_2B_

**FIGURE 1:**
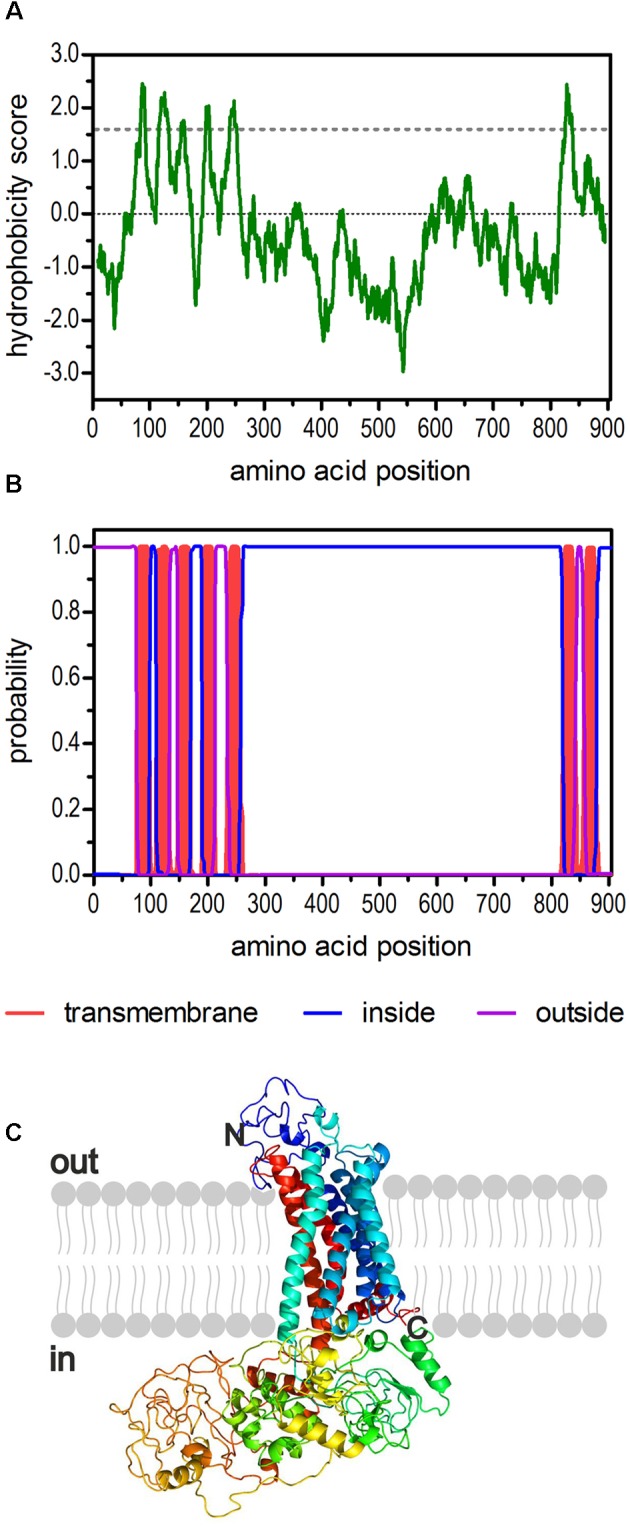
**Structural characteristics of the deduced amino acid sequence of Dm5-HT_2B_.** **(A)** Hydrophobicity profile of Dm5-HT_2B_. The profile was calculated according to the algorithm of Kyte and Doolittle (1982) using a window size of 19 amino acids. Peaks with scores greater than 1.6 (dashed line) indicate possible transmembrane regions. **(B)** Prediction of transmembrane domains with TMHMM server v. 2.0 (Krogh et al., 2001). Putative transmembrane domains are indicated in red. Extracellular regions are shown as purple line, intracellular regions as blue line. **(C)** The primary sequence of Dm5-HT_2B_ was submitted to Phyre2 (Kelley et al., 2015). The 3D model of the receptor is color-coded (rainbow). The extracellular N-terminus and the intracellular C-terminus are labeled.

The sequence of a second potential 5-HT_2_ receptor from *D. melanogaster* had been annotated in previous studies (Brody and Cravchik, 2000; Clark et al., 2004; Hauser et al., 2006). Later, *Dm5-ht2b* (CG42796) was experimentally proven to encode a functional 5-HT receptor (Gasque et al., 2013). Here, we used the available sequence information and applied a PCR-based strategy to amplify the full-length *Dm5-ht2b* cDNA for subsequent detailed pharmacological characterization of this receptor. The *Dm5-ht2b* cDNA contains an open reading frame (ORF) of 2,715 bp and encodes a protein of 904 amino-acid residues (Dm5-HT_2B_) with a calculated molecular mass of 99.5 kDa. The hydrophobicity profile according to Kyte and Doolittle (1982) and prediction of transmembrane helices using TMHMM Server v. 2.0 (Krogh et al., 2001) suggest seven trans-membrane (TM) domains (**Figures 1A,B**), which is a characteristic feature of GPCRs. The TM segments are flanked by an extracellular N-terminus of 74 residues and an intracellular C-terminus of 26 residues. The Dm5-HT_2B_ receptor contains an extremely long third cytoplasmic loop (CPL3) of 563 residues. We submitted the Dm5-HT_2B_ sequence to Phyre2 (Kelley et al., 2015) and obtained a three dimensional-model of the receptor (**Figure 1C**).

**FIGURE 2:**
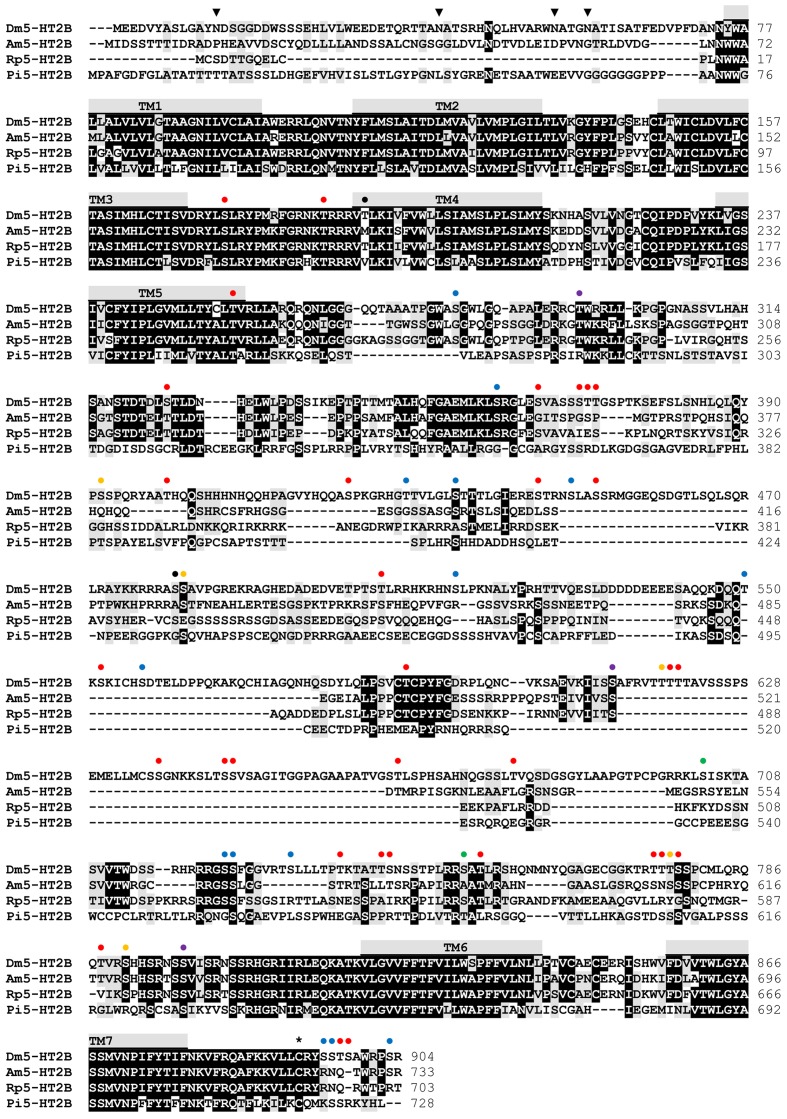
**Amino acid sequence alignment of Dm5-HT_2B_ and orthologous receptors from *Apis mellifera* (Am5-HT_2B_, CBX90121), *Rhodnius prolixus* (Rp5-HT_2B_, AKQ13312), and *Panulirus interruptus* (Pi5-HT_2B_, AAS57919).** Identical residues (≥75%) are shown as white letters against black, whereas conservatively substituted residues are shaded. Putative transmembrane domains (TM1–TM7) are indicated by gray bars. Potential N-glycosylation sites (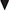), PKA phosphorylation sites (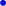), PKC phosphorylation sites (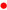), phosphorylation sites for both PKA and PKC (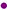), PKG phosphorylation sites (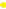), phosphorylation sites for both PKA and PKG (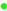), phosphorylation sites for all three kinases (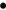), and putative palmitoylation sites (^∗^) of Dm5-HT_2B_ are indicated. The amino acid position is given on the right.

Sequence motifs which are essential for three-dimensional structure, ligand binding, and signal transduction of the receptor are well conserved between the various 5-HT_2B_ receptors (**Figure 2**) and are also present in Dm5-HT_2B_. Three consensus motifs for potential N-glycosylation (N-X-S/T) are located in the extracellular N-terminus of Dm5-HT_2B_ (**Figure 2**). A cysteine residue in the C-terminus (Cys_892_) is a possible site for post-translational palmitoylation. Twenty phosphorylation sites for protein kinase A (PKA), 38 phosphorylation sites for protein kinase C (PKC) and nine phosphorylation sites for protein kinase G (PKG) are present within intracellular domains of Dm5-HT_2B_ (**Figure 2**). N-glycosylation sites were predicted by NetNGlyc 1.0 Server^1^ and putative palmitoylation sites were predicted using GPS-Lipid^2^. Putative phosphorylation sites were predicted by NetPhos 3.1 Server^3^ (Blom et al., 2004).

**FIGURE 3:**
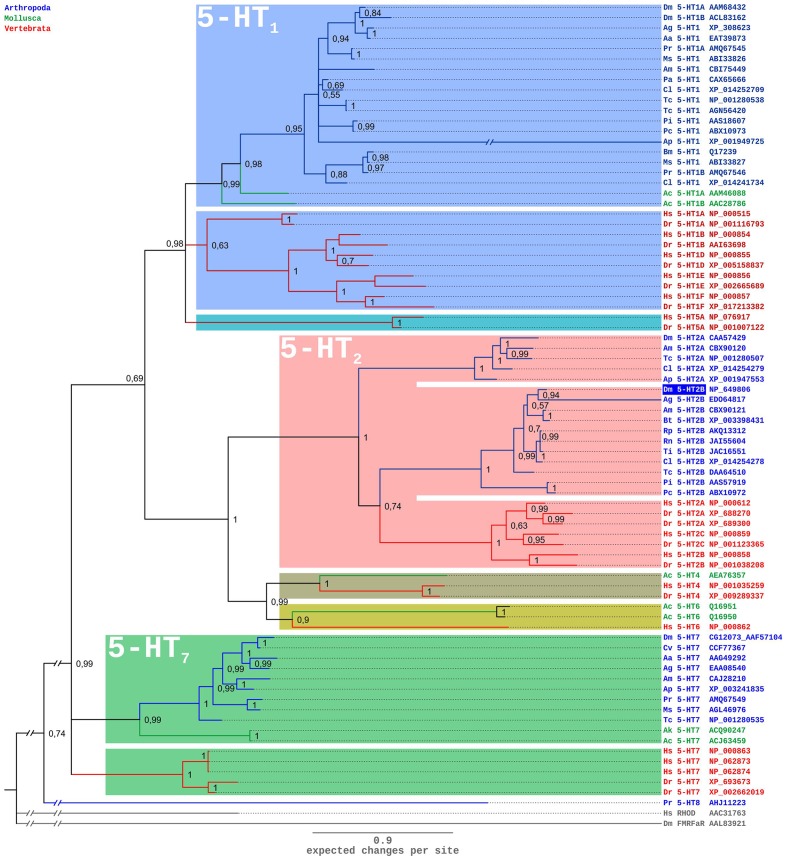
**Bayesian phylogeny of 5-HT receptors.** Alignments were performed using Clustal W (Thompson et al., 1994) by using the core amino-acid sequences lacking the variable regions of the N- and C-terminus and the third cytoplasmic loop. Human rhodopsin (Hs RHOD) and *D. melanogaster* FMRFamide receptor (Dm FMRFaR) were used to root the tree. Numbers at branches represent the posterior probabilities. Receptor subclasses are highlighted by distinct colors. Abbreviations of species in alphabetical order: Aa *Aedes aegypti*, Ac *Aplysia californica*, Ag *Anopheles gambiae*, Ak *Aplysia kurodai*, Am *Apis mellifera*, Ap *Acyrthosiphon pisum*, Bm *Bombyx mori*, Bt *Bombus terrestris*, Cv *Cimex lectularius*, Cv *Calliphora vicina*, Dm *Drosophila melanogaster*, Dr *Danio rerio*, Hs *Homo sapiens*, Ms *Manduca sexta*, Pa *Periplaneta americana*, Pc *Procambarus clarkii*, Pi *Panulirus interruptus*, Pr *Pieris rapae*, Rn *Rhodnius neglectus*, Tc *Tribolium castaneum*, Ti *Triatoma infestans.*

A comparison of the Dm5-HT_2B_ amino-acid sequence with NCBI databases identified several orthologous protostomian and deuterostomian 5-HT_2_ receptors. The highest amino acid identity (ID) and similarity (S) was found with the 5-HT_2B_ receptor of *A. mellifera* (Am5-HT_2B_; Thamm et al., 2013; ID 48.5%, S 58.0%). Homology was also pronounced to 5-HT_2B_ receptors from the kissing bug *R. prolixus* (Rp5-HT_2B_; Paluzzi et al., 2015; ID 44.6%, S 54.1%), and the crustaceans *P. interruptus* (Pi5-HT_2B_; Clark et al., 2004; ID 33.3%, S 45.9%), *Procambarus clarkii* (Pc5-HT_2B_; Spitzer et al., 2008; ID 34.1%, S 46.0%), and *Macrobrachium rosenbergii* (Mr5-HT_2B_; Vázquez-Acevedo et al., 2009; ID 32.8%, S 46.0%). In phylogenetic tree analyses (**Figure 3**), Dm5-HT_2B_ forms a highly supported cluster with other protostomian 5-HT_2B_ receptors. This protostomian 5-HT_2B_ cluster represents the sister group to deuterostomian 5-HT_2_ receptors within a monophyletic 5-HT_2_ receptor group. However, the basal branching of 5-HT-receptor subgroups is not stable and thus has to be subject of future studies.

### Functional and Pharmacological Properties of Dm5-HT_2B_

**FIGURE 4:**
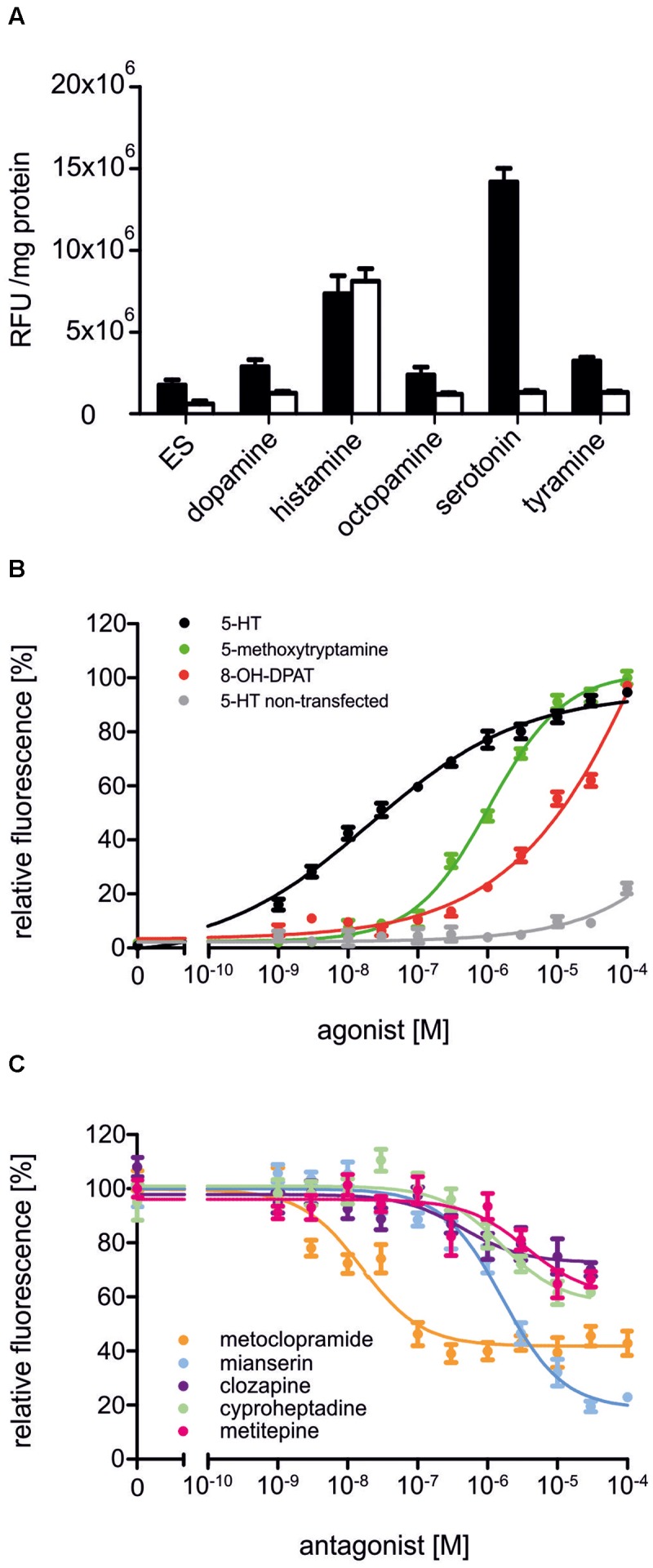
**Pharmacological properties of Dm5-HT_2B_.** **(A)** Effect of different biogenic amines on Ca^2+^-dependent Fluo-4 fluorescence in Dm5-HT_2B_-expressing and non-transfected HEK 293 cells. Bars represent changes in relative fluorescence units (RFU/mg protein) in Dm5-HT_2B_-expressing (black bars) and non-transfected HEK 293 cells (white bars). Biogenic amines were applied in a concentration of 10^-6^ M. Mean values ± SD were calculated from octuplicate determinations. ES, extracellular solution. **(B)** Concentration-dependent effects of serotonin on Dm5-HT_2B_-expressing (black) and non-transfected HEK 293 cells (gray) as well as of 5-methoxytryptamine (green) and 8-OH-DPAT (red) on Dm5-HT_2B_-expressing cells. Data from representative experiments are shown. (Each data point represents the mean ± SD of an octuplicatedetermination. The relative fluorescence signal (%) for measurementswith serotonin was normalized to the value measured in the presenceof 10^-4^ M serotonin in Dm5-HT_2B_-expressing cells (=100%). The relative fluorescence signals (%) for measurements with 5-methoxytryptamine and 8-OH-DPAT were normalized to the value measured in the presence of 10^-4^ M of the respective ligand (=100%). **(C)** Concentration-dependenteffects of potential antagonists on serotonin-stimulated Dm5-HT_2B_-evoked Ca^2+^ signals. Increasing concentrations (10^-9^ M to 10^-4^ M) of receptor antagonists were added to the receptor-expressing cell line. The Ca^2+^-dependent Fluo-4 signals were registered and normalized to thefluorescence evoked with 10^-7^ M serotonin (=100%). Data from representative experiments are shown. Each data point represents the mean ± SD of an octuplicate determination.)

In a first set of experiments, Dm5-HT_2B_-expressing cells and non-transfected HEK 293 cells were incubated with the biogenic amines dopamine, histamine, octopamine, serotonin, and tyramine (1 μM each, **Figure 4A**). The application of serotonin led to an increase in the fluorescence signal in Dm5-HT_2B_-expressing but not in non-transfected cells. Neither dopamine, octopamine nor tyramine evoked responses in transfected or non-transfected cells. Histamine, however, caused a rise in Ca^2+^-dependent Fluo-4 fluorescence in both, Dm5-HT_2B_-expressing and non-transfected HEK 293 cells. This effect is due to endogenously expressed histamine (H1) receptors in the HEK 293 cell line used in this study (Meisenberg et al., 2015).

To further investigate the pharmacological properties of Dm5-HT_2B_, concentration-response curves on Dm5-HT_2B_-expressing and non-transfected HEK 293 cells were established for serotonin. A series of serotonin concentrations was applied ranging from 10^-9^ M to 10^-4^ M. The concentration-response curve for Dm5-HT_2B_ was sigmoid and saturated at a serotonin concentration of 3 × 10^-5^ M (**Figure 4B**). Half-maximal activation of Dm5-HT_2B_ (EC_50_) was at 2.11 × 10^-8^ M. In non-transfected HEK 293 cells, a slight increase in the fluorescence signal was observed at the highest ligand concentration applied (10^-4^ M).

Two potential agonists were tested for their activity on Dm5-HT_2B_-expressing cells. For 5-methoxytryptamine and 8-OH-DPAT, concentration series ranging from 10^-9^ M to 10^-4^ M were applied and Ca^2+^-dependent Fluo-4 fluorescence was monitored (**Figure 4B**). Both ligands caused specific responses. The EC_50_ for 5-methoxytryptamine was 1.05 × 10^-6^ M. In contrast to serotonin and 5-methoxytryptamine, the concentration-response curve for 8-OH-DPAT did not saturate and, therefore, the deduced EC_50_ of ≅6.5 × 10^-4^ M might be taken with some caution.

Next, we examined the ability of potential receptor antagonists for impairing Dm5-HT_2B_ activity. Measurements were performed with increasing concentrations of the antagonists clozapine, cyproheptadine, ketanserin, metitepine (also known as methiothepin), methysergide, metoclopramide, mianserin, prazosin, SB 242084, and spiperone on a background of 10^-7^ M serotonin.

In Dm5-HT_2B_-expressing cells, many of the antagonists caused a decrease of the serotonin-induced Ca^2+^-dependent fluorescence signals. Representative data are shown in **Figure 4C**. Ligand concentrations that led to half-maximal inhibition of Dm5-HT_2B_-induced responses (IC_50_) were determined from the concentration-response curves and are summarized in **Table 1**. The most effective antagonist on serotonin-stimulated Dm5-HT_2B_ was metoclopramide with an IC_50_ of 1.78 × 10^-8^ M. The order of antagonist efficiency (IC_50_) on the Dm5-HT_2B_ receptor was: metoclopramide > clozapine > cyproheptadine > mianserin > metitepine (**Table 1**). Two ligands, prazosin and spiperone, also caused a reduction of the cellular response. However, the signals did not reach saturation and, due to solubility problems higher concentrations could not be tested (see Supplementary Figure S1). Therefore, IC_50_ values were not calculated from these concentration-response curves. For three ligands, i.e., ketanserin, methysergide, and SB 242084, we did not observe any effect on serotonin-stimulated Dm5-HT_2B_-expressing cells.

**Table 1:** IC_50_ values (potency) and relative efficacy were calculated from concentration-response curves for each drug.

	IC_50_ (M)	Log IC_50_ ± SD	Maximal inhibition
Clozapine	4.45 × 10^-7^	-6.35 ± 0.29	40%
Cyproheptadine	1.58 × 10^-6^	-5.80 ± 0.19	35%
Ketanserin	no effect	-	-
Metitepine	3.56 × 10^-6^	-5.45 ± 0.24	35%
Methysergide	no effect	-	-
Metoclopramide	1.59 × 10^-8^	-7.80 ± 0.12	60%
Mianserin	1.64 × 10^-6^	-5.79 ± 0.08	75%
Prazosin	no saturation	-	50%
SB 242084	no effect	-	-
Spiperone	no saturation	-	25%


Efficacy is given as the maximal inhibition (%) of Ca^*2+*^-dependent fluorescence induced by 10^-7^ M serotonin in Dm5-HT_2B_-expressing cells in the absence of antagonist. Values are means of representative experiments in which each data point was obtained from of an octuplicate determination.

## Discussion

There is ongoing interest to precisely understand the physiological and behavioral roles of serotonergic signaling. To meet this challenge, important steps are to determine the molecular and functional-pharmacological properties of 5-HT receptor subtypes and to address their distribution within the CNS. Based on a rich body of data, a picture emerges that, e.g., insects and mammals share similar modes of drug action as well as cellular and behavioral responses to serotonergic neurotransmission. Using model insects such as *D. melanogaster* might accelerate the gain of knowledge. Here, we have focused on elucidating the pharmacological properties of a *D. melanogaster* 5-HT receptor, Dm5-HT_2B_. The pharmacological profile can be used for designing rational *in vitro* and *in vivo* studies to uncover the contribution of Dm5-HT_2B_ to the animal’s development, physiology, and behavior.

### Molecular Features of the Dm5-HT_2B_ Receptor

Four genes encoding 5-HT receptor subtypes were already cloned from *D. melanogaster* in the 90′s of the last century. These were Dm5-HT_1A_ and Dm5-HT_1B_ (CG16720 and CG15113; Saudou et al., 1992), Dm5-HT_2A_ (CG1056; Colas et al., 1995), and Dm5-HT_7_ (CG12073; Witz et al., 1990). These GPCRs share cognate properties with mammalian 5-HT_1A_, 5-HT_2_, and 5-HT_7_ receptors. Resulting from bioinformatics screening and gene annotation, another GPCR gene (CG42796; Brody and Cravchik, 2000; Hauser et al., 2006; Blenau and Thamm, 2011) was uncovered encoding a protein with pronounced similarity to a 5-HT_2_ receptor in the spiny lobster, *P. interruptus* (Clark et al., 2004). The receptor was named Dm5-HT_2B_. In a recent study in which *D. melanogaster* larvae were used to screen for drugs that mediate food intake, the 5-HT receptor antagonist metitepine was identified as a potent anorectic drug (Gasque et al., 2013). Using cell-based assays, the authors could show that metitepine is an antagonist of all five *D. melanogaster* 5-HT receptors including Dm5-HT_2B_ (Gasque et al., 2013). While Gasque et al. (2013) could identify Dm5-HT_2A_ as the sole molecular target for feeding inhibition by metitepine, they did not establish a full pharmacological profile for Dm5-HT_2B_. Here, we provide additional information on the molecular and pharmacological properties of this fifth 5-HT receptor subtype of the fruit fly.

With 904 amino acid residues and a calculated molecular weight of 99.5 kDa, the Dm5-HT_2B_ protein is rather large. More than half of the residues (563 amino acids) are present in the third cytoplasmic loop. Interestingly, the Dm5-HT_2A_ receptor is of similar size and contains 869 amino acid residues (Colas et al., 1995). This receptor also harbors a long third cytoplasmic loop of 321 residues but, in addition, Dm5-HT_2A_ has a long N-terminal loop which consists of 286 residues. For this receptor, two variants have been described. Either a point mutation changing Pro_52_ to Ser in the N-terminus (Schaerlinger et al., 2007) or the complete deletion of the N-terminal domain leads to a significant gain of the receptor’s affinity for serotonin (Colas et al., 1997) compared to the wild type protein. Orthologous receptors to Dm5-HT_2A_ and Dm5-HT_2B_ have been characterized from other insects as well. The Am5-HT_2B_ receptor from the honeybee also contains a large third cytoplasmic loop consisting of 399 residues (Thamm et al., 2013). However, with 80.7 kDa (733 amino acid residues) the protein is smaller than Dm5-HT_2B_. With 653 residues, the honeybee Am5-HT_2A_ receptor is the smallest protein of this foursome. For both honeybee 5-HT_2_ receptor subtypes, several splice variants were molecularly cloned (Thamm et al., 2013). None of these variants gave rise to functional receptors upon heterologous expression of the constructs. This finding, however, does not rule out that full-length and modified variants may assemble in native tissues and thereby potentially expand the repertoire of serotonin binding partners in the honeybee.

Although Dm5-HT_2B_ is set apart by the length of its primary structure from other GPCRs, the protein shares most of the cognate features characterizing this huge gene family. The N-terminus of Dm5-HT_2B_ contains several consensus motifs for post-translational glycosylation (**Figure 2**). A large number of phosphorylation sites to common protein kinases are spread throughout the intracellular loops (**Figure 2**). Which of these sites participate in receptor desensitization and/or internalization (Lefkowitz and Shenoy, 2005; Kelly et al., 2008) upon serotonin stimulation awaits independent experimental testing. In addition to site-directed mutagenesis of single or multiple phosphorylation sites, a deletion strategy might be applied to successively reduce the size of the third cytoplasmic loop connecting transmembrane regions (TM) five and six (**Figure 2**). After heterologous expression of these receptor variants, their signaling properties can be examined and quantified by Ca^2+^ fluorimetry. Finally, residues in the binding site for serotonin that is formed by the transmembrane segments of Dm5-HT_2B_ are well conserved. Notably, the aspartic acid residue (D_153_; D^3.32^; nomenclature to Ballesteros and Weinstein, 1995) in TM3 is a potential binding partner of the protonated amino group of serotonin. A serine residue (S_237_; S^5.43^) in TM5 could bind to the 5-hydroxy group of serotonin’s phenyl moiety. Phenylalanine and/or tryptophan residues in TM6 and TM7 (**Figure 2**) might contribute to π-π interaction with delocalized electrons in serotonin and stabilize the receptor ligand interaction.

Although we haven’t experimentally addressed the expression pattern of the *Dm5-ht2b* gene in this study, compelling evidence is available from previous studies supporting the general finding that 5-HT receptors are widely expressed in the CNS throughout development of *D. melanogaster* (Yuan et al., 2005, 2006; Nichols, 2007). Since we and others (Gasque et al., 2013) have cloned the cDNA encoding Dm5-HT_2B_ from adult tissue, the previous statement also holds for Dm5-HT_2B._ Within the brain of adult flies, Dm5-HT_2B_ is expressed in the pars intercerebralis, the ellipsoid body, and photoreceptor cells (Gnerer et al., 2015). Whether the receptor participates in the regulation of heart function in *D. melanogaster* as suggested by recent experiments (Majeed et al., 2014) or is differentially expressed in male and female nervous tissue (Goldman and Arbeitman, 2007), awaits further testing.

### Pharmacological Properties of Dm5-HT_2B_

The Dm5-HT_2B_ receptor was functionally expressed in HEK 293 cells. Coupling of Dm5-HT_2B_ to intracellular signaling cascades was examined via cell-endogenous G-proteins. Like its protostomian and deuterostomian orthologs, Dm5-HT_2B_ caused intracellular Ca^2+^ release after stimulation with serotonin or synthetic agonists like 5-methoxytryptamine or 8-OH-DPAT. With an EC_50_ of 2 × 10^-8^ M, activation of the receptor was much more sensitive to serotonin compared to 5-methoxytryptamine (EC_50_ ≅1 × 10^-6^ M) or 8-OH-DPAT (EC_50_ ≅6.5 × 10^-4^ M). Since the concentration-response curve with 8-OH-DPAT did not saturate, this latter value should be taken with caution. More recently, two 5-HT_2_ receptors from the honeybee, Am5-HT_2A_ and Am5-HT_2B_, have been molecularly and pharmacologically characterized using the same heterologous expression system (Thamm et al., 2013). With EC_50_ values of 2.57 × 10^-8^ M and 3.25 × 10^-8^ M both receptors share similar potencies for serotonin as Dm5-HT_2B_ and the Cv5-HT_2A_ receptor from *Calliphora vicina* (2.4 × 10^-8^ M; Röser et al., 2012), which was also expressed in HEK 293 cells. With an EC_50_ of 2.01 × 10^-7^ M an orthologous receptor cloned from *R. prolixus* (Rp5-HT_2B_; Paluzzi et al., 2015) was an order of magnitude less sensitive to serotonin. It should be mentioned here, that Rp5-HT_2B_ was not expressed in HEK 293 cells but in a recombinant Chinese hamster ovary cell line (CHOK1-aeq) and that ligand affinity may be influenced by the expression system used. In contrast to Dm5-HT_2B_, where half-maximal stimulation with 5-methoxytryptamine was at ≅1 × 10^-6^ M, both honeybee 5-HT_2_ receptors and the *C. vicina* receptor displayed EC_50_ values in the nanomolar range [Am5-HT_2A_, 7 × 10^-8^ M; Am5-HT_2B_, 6.04 × 10^-8^ M (Thamm et al., 2013); Cv5-HT_2A_, 6.7 × 10^-8^ M (Röser et al., 2012)]. Similar to the results obtained for 5-methoxytryptamine, Dm5-HT_2B_ receptor activation by 8-OH-DPAT (EC_50_ ≅6.5 × 10^-4^ M) was less efficacious than that of Am5-HT_2A_ (EC_50_ = 5.59 × 10^-5^ M) and Am5-HT_2B_ receptors (EC_50_ = 5.6 × 10^-7^ M; Thamm et al., 2013) or the Cv5-HT_2A_ receptor (EC_50_ = 6.2 × 10^-5^ M; Röser et al., 2012). Thus, although active on Dm5-HT_2B_, both 5-methoxytryptamine and 8-OH-DPAT may not serve as alternatives to serotonin in specifically stimulating the receptor since both are likely to activate additional receptor subtypes at concentrations required for *in vivo* application in *D. melanogaster*.

Inhibition of receptor-mediated Ca^2+^ signaling in the cell line constitutively expressing Dm5-HT_2B_ was examined with a series of synthetic antagonists. In addition to substances that completely lacked inhibitory potential on the receptor (i.e., ketanserin, methysergide, and SB 242084), we observed three distinct types of inhibition profiles on Dm5-HT_2B_. Two antagonists caused saturating responses and reduced serotonin-evoked Ca^2+^-dependent fluorescence to values ≤ 40% of control measurements. With an IC_50_ of 1.59 × 10^-8^ M, metoclopramide was more potent than mianserin (IC_50_ = 1.64 × 10^-6^ M). Serotonin-evoked cellular Ca^2+^ responses were reduced to 40 and 25% of control measurements without antagonists by metoclopramide and mianserin, respectively. Responses to clozapine (IC_50_ = 4.45 × 10^-7^ M), cyproheptadine (IC_50_ = 1.58 × 10^-6^ M), and metitepine (IC_50_ = 3.56 × 10^-6^ M) also saturated but all three substances were much less potent inhibitors at the receptor than metoclopramide or mianserin (**Figure 4C**). A maximal reduction to 60% of the serotonin-evoked signal was achieved with clozapine (**Table 1**). Finally, prazosin and spiperone also reduced serotonin-induced Ca^2+^-dependent fluorescence in the cell line but the responses did not saturate. From the whole series of antagonists used in the current study, only metitepine has been tested in an earlier study by Gasque et al. (2013), who expressed Dm5-HT_2B_ in HEK 293T cells to investigate the pharmacology of this drug on *D. melanogaster* 5-HT receptors. Using Ca^2+^ fluorimetry on individual cells expressing Dm5-HT_2B_, the authors reported an IC_50_ of 2 × 10^-6^ M which is very similar to the value determined in the current study. Interestingly, metitepine has been uncovered as a potent anorectic drug in *D. melanogaster* larvae (Gasque et al., 2013). Although active on all five 5-HT receptor subtypes of the fruit fly, metitepine exhibited its anti-feeding activity only by interfering with Dm5-HT_2A_ signaling (Gasque et al., 2013). Some of the antagonists tested on Dm5-HT_2B_ in the current study had been examined previously on honeybee, *C. vicina*, and *R. prolixus* 5-HT receptors, too. Clozapine, cyproheptadine, metitepine, and mianserin inhibited Am5-HT_2A_ receptors in the micromolar range and reduced serotonin-induced Ca^2+^-dependent fluorescence by 44, 36, 39, and 49%, respectively (Thamm et al., 2013). Interestingly, at the Am5-HT_2B_ receptor metitepine did not have any activity at all. In contrast, clozapine, cyproheptadine, and mianserin blocked Ca^2+^-dependent fluorescence to 5, 23, and 24%, respectively, with IC_50_ values in the low micromolar range (Thamm et al., 2013). Efficient inhibitors acting on the blowfly Cv5-HT_2A_ receptor were metitepine and clozapine which reduced serotonin-induced Ca^2+^ signals to 15 and 25% of control measurements with IC_50_ values of 1.2 × 10^-6^ M and 15 × 10^-6^ M, respectively (Röser et al., 2012). Cyproheptadine, ketanserin, and mianserin reduced activity of the *R. prolixus* Rp5-HT_2B_ receptor by ≥ 50% at the highest from three concentrations tested, i.e., 10^-7^, 10^-6^, and 10^-5^ M (Paluzzi et al., 2015).

In the current study, we identified metoclopramide as the most potent antagonist at the Dm5-HT_2B_ receptor. This was surprising, since metoclopramide is an established dopamine D_2_ receptor antagonist in vertebrates, where it also inhibits serotonin-gated ion channels (5-HT_3_ receptors) and activates 5-HT_4_ receptors (Tonini et al., 1995). The latter effects have been therapeutically used to interfere with emesis. Until now, no information is available regarding the pharmacology of metoclopramide on the remaining four *D. melanogaster* 5-HT receptors. Future studies must show whether metoclopramide is really a Dm5-HT_2B_-specific or a rather non-selective antagonist of 5-HT receptors in the fruit fly. Metoclopramide has also been shown to bind to the tyramine receptor TyrR (CG7431; Arakawa et al., 1990; *K*_i_ = 4.6 × 10^-6^ M) and to block the β-adrenergic-like octopamine receptor Octβ3R (CG42244; Maqueira et al., 2005) in *D. melanogaster*, although only a high concentration of 10^-5^ M was tested in the latter study. Due to its pronounced sensitivity to Dm5-HT_2B_ (IC_50_ = 1.59 × 10^-8^ M), however, the concentration required for *in vivo* experimentation to specifically target this receptor subtype might be kept rather low. In summary, our data may facilitate future behavioral pharmacological studies on the role of Dm5-HT_2B_ in the fruit fly. Such studies would be desirable, since current knowledge on the role of this 5-HT receptor subtype solely depend on the investigation of flies that have been genetically manipulated.

## Author Contributions

WB designed and evaluated experiments, wrote the paper; DS conducted experiments and evaluated data; SB conducted experiments and evaluated data; MT conducted experiments; AB designed experiments and wrote the paper.

## Conflict of Interest Statement

The authors declare that the research was conducted in the absence of any commercial or financial relationships that could be construed as a potential conflict of interest.

## Footnotes

1 http://www.cbs.dtu.dk/services/NetNGlyc/

2 http://lipid.biocuckoo.org/

3 http://www.cbs.dtu.dk/services/NetPhos/
