# Supplementary material for: Dm5-HT2B: Pharmacological Characterization of the Fifth Serotonin Receptor Subtype of Drosophila melanogaster
Source: Front Syst Neurosci. 2017 May 11;11:28. doi: 10.3389/fnsys.2017.00028 (PMC5425475; doi:10.3389/fnsys.2017.00028)
Supplement: Supplementary file 1 [file Image_1.PDF]

Figure S1

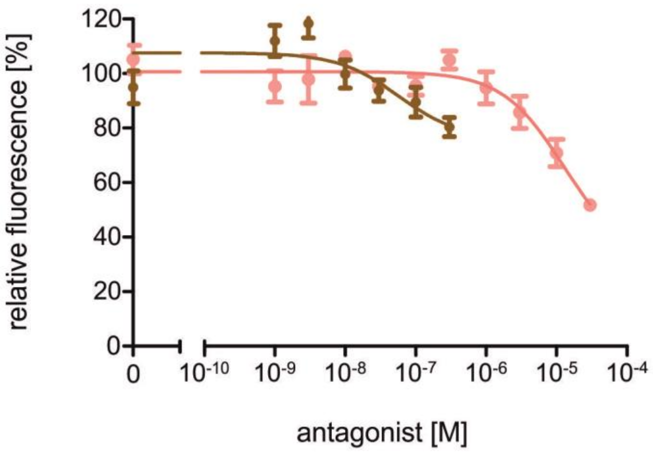

Concentration-dependent effects of potential antagonists on serotonin-stimulated Dm5-HT_{2B}-evoked Ca^{2+} signals. Dm5-HT_{2B}-expressing cells were loaded with the Ca^{2+}-sensitive dye Fluo-4. Increasing concentrations of prazosin (pink) or spiperone (brown) were added to the receptor-expressing cell line. The Ca^{2+}-dependent Fluo-4 signals were registered and normalized to the fluorescence evoked with 0.1 µM serotonin (= 100%). A representative experiment is displayed. Each data point represents the mean ± SD of an eight-fold determination. Both ligands inhibited Dm5-HT_{2B} receptor activity but the responses did not saturate.
